# Resistance Index of the Superior Mesenteric Artery: Correlation With Lactate Concentration and Kinetics Prediction After Cardiac Surgery

**DOI:** 10.3389/fmed.2021.762376

**Published:** 2021-11-24

**Authors:** Yuankai Zhou, Huaiwu He, Xiaoting Wang, Na Cui, Xiang Zhou, Yun Long, Dawei Liu

**Affiliations:** State Key Laboratory of Complex Severe and Rare Diseases, Department of Critical Care Medicine, Peking Union Medical College, Peking Union Medical College Hospital, Chinese Academy of Medical Sciences, Beijing, China

**Keywords:** post-cardiac surgery, superior mesenteric artery, end-diastolic velocity, resistance index, lactate kinetics, lactate

## Abstract

**Objective:** This study aimed to measure blood flow changes in the superior mesenteric artery (SMA), using Doppler ultrasound, in post-cardiac surgery patients, to evaluate the correlation between the SMA resistance index (SMA-RI) and lactate concentrations.

**Methods:** The patients' basic hemodynamics, blood gas parameters and lactate concentration were collected at admission. Simultaneously, the SMA blood flow parameters were collected using Doppler ultrasound with the patients in the supine position. The lactate concentrations were measured again at 2, 6, and 12-h time points after the first test. The length of intensive care unit stays and prognoses continued to be monitored.

**Results:** A total of 67 patients were included. The SMA-RI correlated with the admission (*r* = 0.3117, *P* = 0.0102), 2-h (*r* = 0.5091, *P* < 0.0001), 6-h (*r* = 0.5061, *P* < 0.0001), and 12-h (*r* = 0.2483, *P* = 0.0428) lactate concentrations. The SMA-RI could predict the 2-h 10% [area under the curve (AUC) = 0.8294, *P* < 0.0001] and 6-h 40% lactate kinetics (AUC = 0.7708, *P* = 0.0012). The cut-off value was 0.83. When the SMA-RI was <0.83, the specificity and sensitivity were 86.38 and 75.56%, respectively for the prediction of the 2-h >10% lactate kinetics, and 64.71 and 75.00%, respectively, for the prediction of the 6-h >40% lactate kinetics. The lactate concentrations at admission, 2 and 6-h points were higher in the high-RI group (RI ≥ 0.83) and the intensive care unit stays were significantly longer than in the low-RI group (*P* = 0.0005).

**Conclusions:** The increase in SMA-RI was associated with higher lactate concentrations and worse lactate kinetics in post-cardiac surgery patients. This may be related to intestinal hypoperfusion. The SMA-RI may be one of the indicators that should be monitored to guide resuscitation in these patients.

## Introduction

Hyperlactatemia often occurs in patients following cardiac surgery and the mechanisms are often various which include the tissue hypoperfusion, hypothermia, undergoing cardiopulmonary bypass (1, 2). The persistence of hyperlactatemia is associated with a worse prognosis (2–5). In those patients with tissue hypoperfusion, the longer duration of hyperlactatemia always means the longer hypoxia of tissue and greater damage to the organs. Therefore, lactate kinetics should be one of the most vital indicators that guides hemodynamic treatment after cardiac surgery. Currently, there is a lack of indicator that can predict it. This can lead to the delay of treatment, prolonged hyperlactatemia, and a worsening of the prognosis. Thus, we hope to find the indicator of organ hypoperfusion to initiate our treatment in advance to reduce the duration of hypoperfusion, so as to improve the prognosis of patients after cardiac surgery with tissue hypoperfusion.

The visceral system, including the intestinal tract, receives nearly 25% of the cardiac output and is considered to be the significant blood storage sites. Therefore, monitoring blood flow of mesenteric organs may be a potential method that can be used to assess resuscitation status, which may affect the lactate kinetics directly (6, 7).

The blood blow in the superior mesenteric artery (SMA) provides the largest blood supply to the small intestine and thus can be representative. Color-coded Doppler ultrasound, a convenient and non-invasive technique, can be used for monitoring changes in the blood flow in the SMA (8, 9).

The resistance index (RI) is a blood flow parameter calculated using the flow velocity on Doppler imaging and can reflect the resistance of the entire distal intestinal circulation (10, 11). In patients with intestinal microcirculation disorders of various causes, the arterial resistance increases, resulting in a higher SMA-RI. This parameter may provide a “window” to monitor the intestinal tissue perfusion during point-of-care testing.

This study aimed to verify the high intestinal arteriolar vascular bed resistance as the predictor of poor lactate kinetics, which may help the physicians to screen out those patients after cardiac surgery with hypoperfusion of visceral organs.

## Materials and Methods

### Patient Enrolment

This study was approved by the Institutional Research and Ethics Committee of the Peking Union Medical College Hospital (ZS-2105). Written informed consent was obtained from the patients or their next-of-kin prior to enrolment.

This prospective study was conducted at the Critical Care Department of Peking Union Medical College Hospital in China from May 2020 to September 2020.

**Figure 1:**
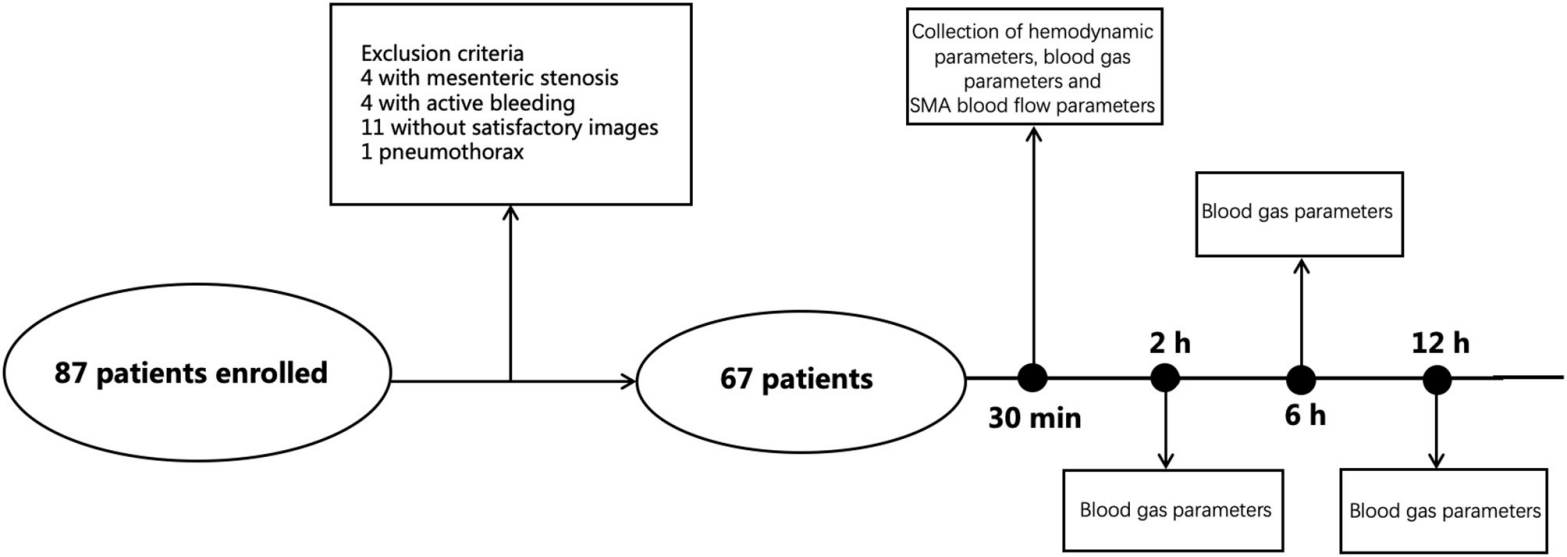
Enrolment flowchart of the study patients.

Patients aged 18–80 years who were admitted to the intensive care unit (ICU) after cardiac surgery were included in this study. The exclusion criteria were: patients with severe stenosis [SMA peak systolic velocity (SMA-PSV) of >275 cm/s, or SMA-PSV/abdominal aorta peak velocity of >3 (12)], active bleeding or pneumothorax during the observation period (which could affect the circulation), hepatic dysfunction, liver cirrhosis, portal hypertension, cancer, and end-stage renal diseases. Moreover, patients who were pregnant and those who underwent procedures involving the descending thoracic aorta were also excluded. During the study period, 87 post-cardiac surgery patients that were admitted to our department, were enrolled (Figure 1).

### Study Design

The patients' basic hemodynamics, blood gas levels, and SMA blood flow parameters were collected after ICU admission. Doppler ultrasound was performed using a 2–5 MHz C60xp Probe (X-Porte Ultrasound System, FUJIFILM Sonosite, Inc., USA). With the patient in the supine position, the SMA flow was measured 1–2 cm proximal to the vessel. The angle of insonation was <60°. The SMA blood flow parameters were measured by two experienced critical care attending physicians, and the average value was recorded. In the case of arrhythmias such as atrial fibrillation, five cardiac cycles were measured, and the subsequent average value was recorded. The time–velocity waveform readings were used to measure the PSV and end-diastolic velocity (EDV). The RI was calculated according to the following formula:


$$RI=\frac{\left(PSV-EDV\right)}{PSV}$$

The lactate concentration was measured at admission, as well as at 2, 6, and 12 h. The kinetics were then calculated according to the following formula (13):


$$Lactate\;kinetics=\frac{Lactate_{initial}-Lactate_{time}}{Lactate_{initial}}\;\times 100\%$$

where Lactate_*initial*_ was the concentration at admission and Lactate_*time*_ the concentration at the relevant time points. These data were analyzed to explore the relationships between SMA-RI, lactate concentration and kinetics in the early postoperative stages of cardiac surgery.

All the selected patients were deprived of food and water for 12 h before cardiac surgery and continued this state until the study finished.

### Statistical Analysis

A descriptive analysis was performed. All the data were presented as mean ± SD or median (25–75th percentile) unless otherwise specified. Continuous variables were analyzed using the *t*-test, analysis of variance, the Mann–Whitney *U*-test, or the Kruskal–Wallis test based on the data distribution and the number of variables. Two continuous variables were compared using linear regression. The discrimination of values was examined using receiver operating characteristic (ROC) analysis. The chi-squared test (or Fisher's exact test when appropriate) was used to compare discrete variables. All comparisons were two-tailed, and a *P*-value of < 0.05 was required to exclude the null hypothesis. All statistical analyses were performed using Prism 8 (GraphPad Software, La Jolla, CA, USA).

## Results

### Demographic and Clinical Characteristics

In total, 67 patients were included in this study. The average age was 58.33 ± 12.29 years. In terms of surgeries, 32.84% of patients (*n* = 22) had undergone coronary artery bypass grafting (CABG), 56.72% (*n* = 38) valvular surgery, 4.48% (*n* = 3) CABG plus valvular surgery, and 4.48% (*n* = 3) other procedures. The average cardiopulmonary bypass and cross-clamp times were 108.26 ± 29.75 min and 75.96 ± 23.55 min, respectively. The sequential organ failure assessment score at the time of enrollment was 9.29 ± 2.80. A total of 95.52% of the enrolled patients required catecholamine support, and the average dose of norepinephrine was 0.19 ± 0.23 μg/kg/min. The patients' average velocity time interval measured using ultrasound was 14.30 ± 4.46 cm/s. The demographic and clinical characteristics of all patients are shown in Table 1.

**Table 1:** Characteristics of patients.

**Variable**	**Frequency (percentage) or mean ± SD**
Number of patients	67
Sex, *n*, female/male	24/43
Age (years)	58.33 ± 12.29
Body Mass Index (kg/m^2^)	22.95 ± 1.94
EF-preop (%)	53.8 ± 10.3
MAP (mmHg)	84.06 ± 12.16
HR (bpm)	92.72 ± 14.46
CVP (mmHg)	7.98 ± 2.06
HGB (g/L)	99.47 ± 33.08
ScvO2 (%)	70.66 ± 8.14
Pv-aCO2 (mmHg)	4.93 ± 1.90
Catecholamine, *n* (%)	64 (95.52)
NE (μg/kg/min)	0.19 ± 0.23
E (μg/kg/min)	0.04 ± 0.06
VTI (cm/s)	14.30 ± 4.46
Perfusion index	1.36 ± 1.21
SOFA	9.29 ± 2.80
APACHE-II	16.06 ± 4.08
EURO-II	3.71 ± 3.25
CPB (min)	108.26 ± 29.75
Cross-clamp time (min)	75.96 ± 23.55
Mechanical Ventilation, *n* (%)	67 (100)
CRRT, *n* (%)	18 (26.9)
ICU LOS (days)	7.27 ± 4.14
28-day mortality, *n* (%)	4 (6)
**Procedures**	
CABG surgery	22 (32.84)
Valve surgery	38 (56.72)
CABG + valve surgery	3 (4.48)
Other	3 (4.48)

*MAP, mean arterial pressure; HR, heart rate; CVP, central venous pressure; HGB, hemoglobin; ScvO_2_, superior vena cava oxygen saturation; Pv-aCO_2_, arterial and venous carbon dioxide partial pressure difference; NE, norepinephrine; E, adrenaline; VTI, velocity time interval; SOFA, Sequential Organ Failure Assessment; APACHE-II, acute physiology and chronic health evaluation scoring system-II; CPB, cardiopulmonary bypass; CRRT, continuous renal replacement therapy; LOS, length of stay; CABG, coronary artery bypass grafting*.

### SMA-RI

The median SMA-RI was 0.86 (0.787–0.889). The distribution is shown in Supplementary Figure 1.

### Correlation Analysis of Lactate Concentrations, SMA Flow, and Hemodynamic Parameters

The median values of the lactate concentrations on admission and after 2 h were 4.10 mmol/L (3.10–6.10 mmol/L) and 4.40 mmol/L (3.00–6.60 mmol/L), respectively. The concentrations decreased to 3.20 mmol/L (2.10–4.90 mmol/L) and 1.90 mmol/L (1.40–2.90 mmol/L) at the 6- and 12-h marks, respectively. The calculated lactate kinetics at each time point are shown in Supplementary Table 1.

**Figure 2:**
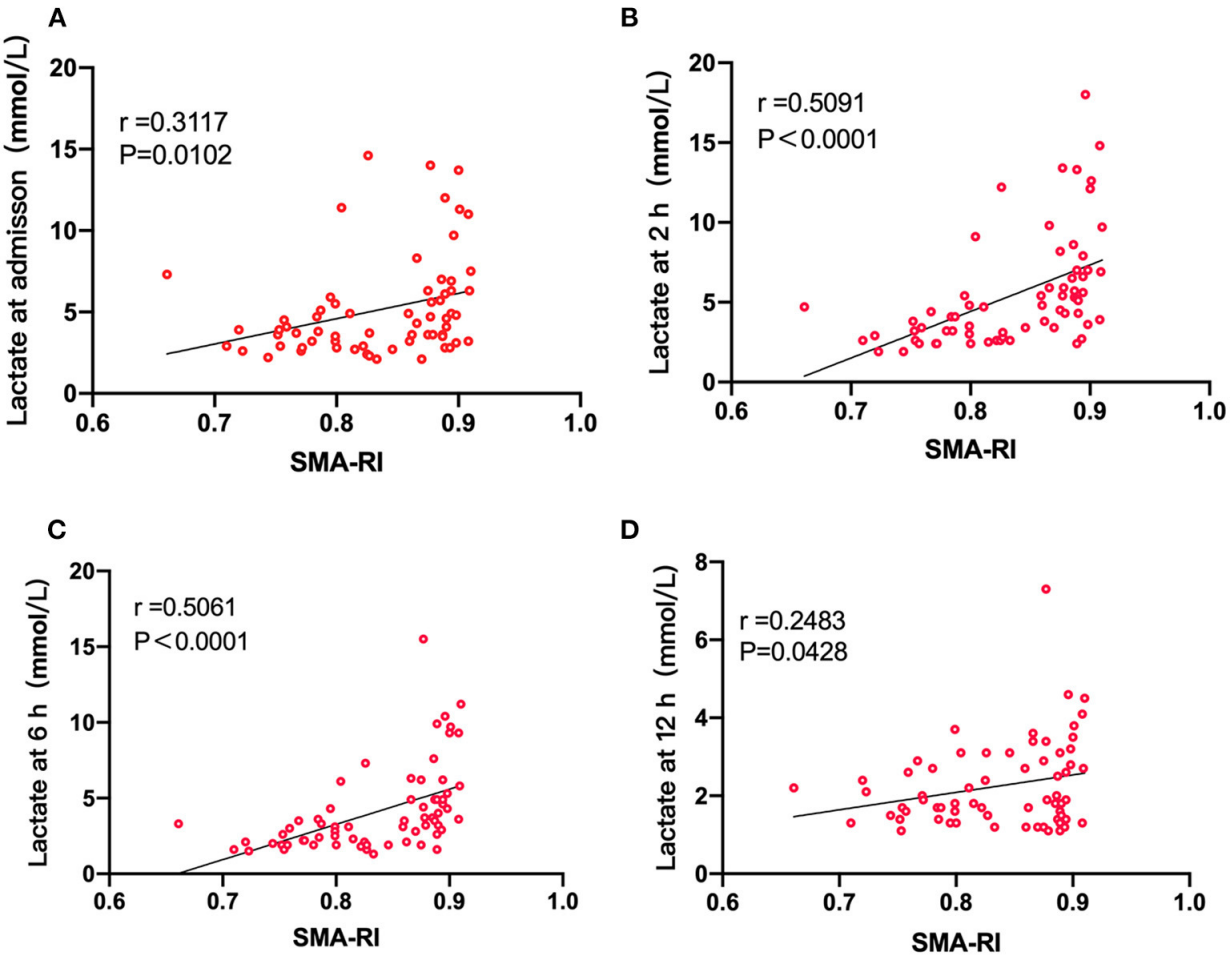
**(A)** The correlation between SMA-RI and the lactate concentration at admission (*r* = 0.3117, *P* = 0.0102). **(B)** The correlation between SMA-RI and the 2-h lactate concentration (*r* = 0.5091, *P* < 0.0001). **(C)** The correlation between SMA-RI and the 2-h lactate concentration (*r* = 0.5061, *P* < 0.0001). **(D)** The correlation between SMA-RI and the 12-h lactate concentration (*r* = 0.2483, *P* = 0.0428).

The SMA-RI was associated significantly with the lactate concentration at admission (*r* = 0.3117, *P* = 0.0102, Figure 2A). A good correlation was noted with the 2-h (*r* = 0.5091, *P* < 0.0001, Figure 2B) and 6-h lactate concentrations as well (*r* = 0.5061, *P* < 0.0001, Figure 2C). Furthermore, a significant correlation was also noted with the 12-h lactate concentration (*r* = 0.2483, *P* = 0.0428, Figure 2D).

The SMA-EDV was significantly and negatively associated with the 2-h (*r* = −0.4085, *P* = 0.0006), 6-h (*r* = −0.4587, *P* = 0.0001), and 12-h lactate concentrations (*r* = −0.3103, *P* = 0.0106), post-cardiac surgery. However, the SMA-PSV, arterial and venous carbon dioxide partial pressure difference (Pv-aCO_2_), superior vena cava oxygen saturation (ScvO_2_), and perfusion index (PI) at admission were not associated with the lactate concentrations at any point (Table 2).

**Table 2:** Correlation analysis of lactate concentrations, superior mesenteric artery flow, and hemodynamic parameters.

**Variable**	**Admission lac**		**2-h lac**		**6-h lac**		**12-h lac**	
	***r***	***P***	***r***	***P***	***r***	***P***	***r***	***P***
SMA-RI	0.3117	0.0102^*^	0.5091	<0.0001^*^	0.5061	<0.0001^*^	0.2483	0.0428^*^
SMA-PSV	0.1774	0.1510	0.1367	0.2699	0.0228	0.8548	−0.1473	0.2342
SMA-EDV	−0.2058	0.0947	−0.4085	0.0006^*^	−0.4587	0.0001^*^	−0.3103	0.0106^*^
Pv-aCO_2_	−0.1520	0.2193	−0.0794	0.5231	−0.0470	0.7055	−0.0201	0.8720
ScvO_2_	0.0938	0.4501	0.0540	0.6644	0.0490	0.6937	−0.0047	0.9696
PI	−0.1977	0.1087	−0.1805	0.1438	−0.1553	0.2095	−0.1631	0.1872

* *P < 0.05; Lac, Lactate concentration; SMA-RI, resistance index of the superior mesenteric artery; SMA-PSV, peak systolic velocity of the superior mesenteric artery; SMA-EDV, end-diastolic velocity of the superior mesenteric artery; Pv-aCO_2_, arterial and venous carbon dioxide partial pressure difference; ScvO_2_, superior vena cava oxygen saturation; PI, Perfusion Index*.

### Correlation Analysis of Lactate Kinetics, SMA Flow, and Hemodynamic Parameters

The SMA-RI was negatively associated with the 2-h (*r* = −0.6074, *P* < 0.0001) and 6-h lactate kinetics (*r* = −0.5266, *P* < 0.0001) while the SMA-EDV showed a positive association (*r* = 0.5829, *P* < 0.0001 and *r* = 0.5490, *P* < 0.0001, respectively). No significant correlations were found between the lactate kinetics and the Pv-aCO_2_, ScvO_2_, or PI (Table 3).

**Table 3:** Correlation analysis of lactate kinetics, superior mesenteric artery flow, and hemodynamic parameters.

**Variable**	**2-h lactate kinetics**		**6-h lactate kinetics**		**12-h lactate kinetics**	
	***r***	***P***	***r***	***P***	***R***	***P***
SMA-RI	−0.6074	<0.0001^*^	−0.5266	<0.0001^*^	0.1815	0.1416
SMA-PSV	0.0464	0.7091	0.1704	0.1681	0.2816	0.0210^*^
SMA-EDV	0.5829	<0.0001^*^	0.5490	<0.0001^*^	−0.0401	0.7473
Pv-aCO_2_	−0.2195	0.0743	−0.2242	0.0682	−0.2159	0.0793
ScvO_2_	0.1633	0.1868	0.1193	0.3362	0.1311	0.2902
PI	−0.1550	0.2103	−0.1100	0.3756	−0.0475	0.7028

* *P < 0.05; SMA-RI, resistance index of the superior mesenteric artery; SMA-PSV, peak systolic velocity of the superior mesenteric artery; SMA-EDV, end-diastolic velocity of the superior mesenteric artery; Pv-aCO_2_, arterial and venous carbon dioxide partial pressure difference; ScvO_2_, superior vena cava oxygen saturation; PI, Perfusion Index*.

### Lactate Kinetics Prediction of >10% at the 2-h and >40% at the 6-h Mark

**Figure 3:**
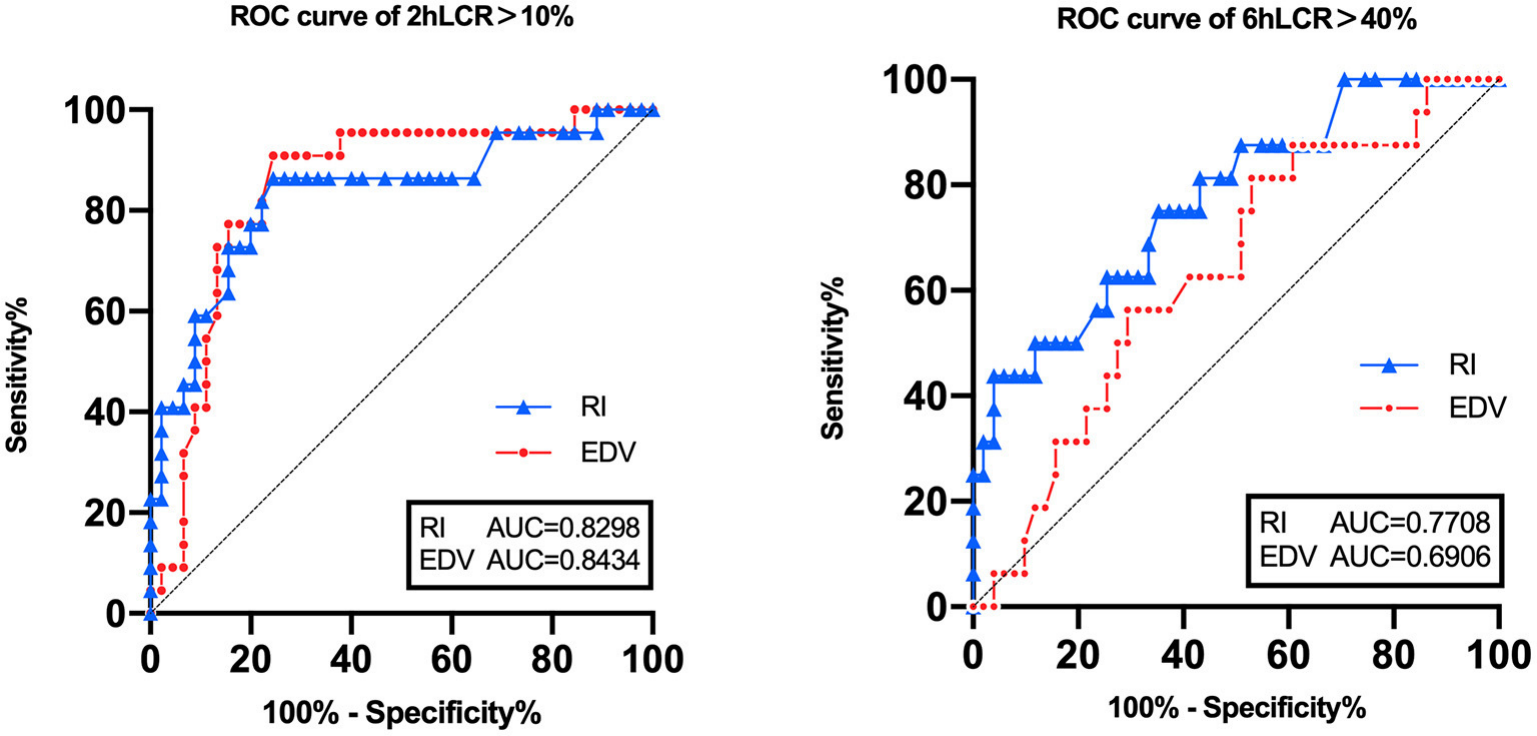
Receiver operating characteristic curves comparing the lactate kinetics' ability to predict superior mesenteric artery resistance index and end-diastolic velocity.

The areas under the curve (AUC) of the SMA-RI and SMA-EDV were used for this prediction. The ROC curves are shown in Figure 3 and the SMA-RI was shown to better predict both. The SMA-RI threshold of 0.83 was associated with the best Youden index for both the 2- and 6-h lactate kinetics curves (Table 4).

**Table 4:** Receiver operating characteristic curve analysis of superior mesenteric artery resistance index and end-diastolic velocity in predicting lactate kinetics.

**Lactate kinetics**	**Variable**	**ROC area**	***P***	**95% CI**	**Cut-off value**	**Sensitivity %**	**Specificity %**
2-h	SMA-RI	0.8298	<0.0001^*^	0.7160–0.9436	0.83	86.38	75.56
>10%	SMA-EDV	0.8434	<0.0001^*^	0.7409–0.9459	19.70 cm/s	81.82	77.78
6-h	SMA-RI	0.7708	0.0012^*^	0.6391–0.9026	0.83	75.00	64.71
>40%	SMA-EDV	0.6906	0.0222^*^	0.7409–0.9459	16.25 cm/s	87.50	50.98

* *P < 0.05 ROC, Receiver operating characteristic; SMA-RI, resistance index of the superior mesenteric artery; SMA-EDV, end-diastolic velocity of the superior mesenteric artery; CI, confidence interval*.

### Clinical Classification Based on the SMA-RI

Based on the preset principle, the patients were assigned to the high-RI (RI ≥ 0.83) and the low-RI (RI < 0.83) groups. The characteristics of the patients between the two groups are shown in Supplementary Table 2. The lactate concentrations and kinetics at all the relevant time points in the two groups are shown in Table 5. The low-RI group exhibited significantly lower lactate concentrations (Supplementary Figure 2) as well as lower lactate kinetics at admission, the 2- and 6-h marks. Ultimately, patients in the low-RI group had a significantly shorter ICU stays than those in the high-RI group [4.00 days (3.00–7.00 days) vs. 8.00 days (5.5–11.00 days), *P* = < 0.0001] (Table 5).

**Table 5:** Lactate concentrations and kinetic changes between the high and low resistance index groups.

**Variables**	**High-RI RI ≥ 0.83 (*n* = 37)**	**Low-RI RI <0.83 (*n* = 30)**	***P***
Admission LAC (mmol/L)	4.90 (3.55–6.95)	3.65 (2.80–4.75)	0.0133^*^
2-h LAC (mmol/L)	5.70 (4.30–8.40)	3.15 (2.58–4.18)	<0.0001^*^
6-h LAC (mmol/L)	4.40 (3.15–6.25)	2.25 (1.90–3.15)	<0.0001^*^
12-h LAC (mmol/L)	2.00 (1.35–3.30)	1.75 (1.50–2.40)	0.2867
2-h lactate kinetics (%)	−14.60 (−29.60–4.85)	12.70 (5.75–18.23)	<0.0001^*^
6-h lactate kinetics (%)	11.10 (−8.40–30.85)	35.95 (19.80–45.05)	<0.0001^*^
12-h lactate kinetics (%)	57.10 (42.90–67.05)	51.80 (32.03–64.98)	0.2009
ICU LOS (days)	8.00 (5.5–11.00)	4.00 (3.00–7.00)	<0.0001^*^
28-day mortality (*n*/%)	3 (10)	1 (2.7)	0.3179

* *P < 0.05. Data are presented as median (interquartile range) except for mortality (number of deaths/percentage). RI, resistance index; LAC, lactate; LOS, length of stay*.

## Discussion

To the best of our knowledge, this study is the first to investigate the SMA-RI and lactate kinetics in post-cardiac surgery patients. We found a good correlation between the SMA-RI and lactate concentrations at admission and the 2, 6, and 12-h time points. Moreover, the SMA-RI was shown to predict the 2-h >10% lactate kinetics and 6-h >40% lactate kinetics at an early stage, with the cut-off value being 0.83. The lactate concentrations at admission and at all the time points were higher in the high-RI group, as was the length of ICU stay, compared to the low-RI group.

### Correlation Between the SMA-RI and Lactate Concentrations

We observed that the SMA-RI in the early phase correlated with the lactate concentration at the 12-h mark. The increase in the SMA-RI could reflect the degree of intestinal perfusion disruption, similar to the renal resistive index, which has been used in the diagnosis of chronic allograft nephropathy and renal artery stenosis (14).

Compared with the 2- and 6-h lactate concentrations, the SMA-RI had a weak correlation at admission. Many intraoperative factors could affect the lactate concentration (2). The correlation between the SMA-RI and the 12-h lactate concentration was also low as various treatments were administered to the patients within this time, resulting in varying confounding factors. Moreover, the lactate concentrations in some patients returned to the reference range before the 12-h postoperative mark and did not continue to decline, which could also affect the correlation.

Lactate concentration and kinetics are important indices to predict the prognosis of critically ill patients (13, 15). They are also important indicators of complications and recovery after cardiac surgery (3, 16, 17). Since hyperlactatemia is a consequence of tissue hypoxia, their use may cause the delay of treatment, which negatively affects early detection of inadequate resuscitation and subsequent organ function. Therefore, the correlation between the SMA-RI and lactate concentrations suggests that intestinal hypoperfusion status may be related to postoperative hyperlactatemia. The hypoperfusion of intestine reflected by the increase of SMA-RI may be one of the treatment directions to reduce the level of lactate concentration after operation.

The SMA-EDV was also correlated with the lactate concentrations at the 2- and 6-h marks. This may be due to the fact that the diastolic blood flow velocity not only reflects high distal vascular resistance but also the average blood flow velocity of each cardiac cycle, reflecting the hypoperfusion of the SMA microcirculation.

### The SMA and Lactate Kinetics

In our study, the SMA-RI and SMA-EDV were found to predict the 2 and 6-h lactate kinetics. Moreover, the closer the lactate kinetics calculation is to the time of blood flow examination, the more accurate the prediction. This phenomenon may also confirm our hypothesis. In practice, the lactate concentration was monitored to examine the resuscitation. After adjusting the treatment, it was measured again after a few hours and the lactate kinetics was recalculated. However, this requires waiting several hours, which could delay treatment and extend the period of organ hypoperfusion.

Measuring SMA-RI may aid the prediction of lactate kinetics in the following 6 h after cardiac surgery. So that the patient with splanchnic organ hypoperfusion can be treated promptly. At the same time, the real-time changes in the SMA flow can be monitored to evaluate the effect of the treatment. This may shorten the duration of hypoxia and resuscitation.

### Comparison of the Classification Based on SMA-RI

To confirm our hypothesis, the patients were divided into high-RI and low-RI groups using a cut-off value of 0.83, based on the AUC of lactate kinetics prediction. The results showed that the lactate concentration was higher in the high-RI group at admission, and the ICU stay was longer. This demonstrates the significance of SMA-RI in examining the recovery of patients after cardiac surgery. Owing to the correlation between the SMA-RI and lactate kinetics, we are able to not only monitor but also to attempt to adjust the SMA-RI by altering the treatment to improve lactate kinetics.

### Systemic Resuscitation and Organ Perfusion

The treatment of shock is changing from systemic to specific organ perfusion monitoring and correction (18), which are expected to improve resuscitation and prognosis. The ScvO_2_, Pv-aCO_2_, and other systemic parameters are not sensitive enough to reflect the hypoperfusion of specific organs. Therefore, ICU physicians require other indicators related to lactate kinetics. As mentioned previously, the mesenteric organs are important blood storage sites, and monitoring the intestinal circulation is an important part of hemodynamic observation.

The SMA is a high-resistance vessel due to its very long branches. The SMA flow is normally laminar, characterized by a three-phase wave composed of a forward peak in the systolic, a backward wave in the early diastolic, and a low-speed forward flow in the middle and late diastolic periods (Supplementary Figure 3).

The SMA-RI may be affected by various factors, providing numerous methods for its reduction. For example, blood flow reduction and increase in the SMA-RI caused by cardiogenic shock may be corrected using cardiac agents. Arginine vasopressin in large doses may be replaced with vasoactive drugs that have smaller negative effects on the intestinal blood flow. During severe stress, adequate sedation and dexmedetomidine may be used. In abdominal hypertension, ascites drainage and gastrointestinal drainage may reduce the intra-abdominal pressure and the SMA-RI. It is also important to note that when the factors causing the RI increase cannot be altered, part of correction may be obtained using other methods. However, these must be verified by follow-up clinical studies and future research.

This study had some limitations. First, the SMA-RI reflected the relationship between peak systolic and end-diastolic blood flow and therefore may only partially reflect the overall state. More indicators, including the pulsatility index need to be considered and further studies are warranted. Second, although the SMA-RI could be affected by many factors, such as vasoactive drugs, vascular elasticity, cardiac output, and intra-abdominal pressure, the accumulation of various factors of intestinal circulation vascular resistance may lead to non-obstructive intestinal ischemia in some patients. Thus, our study only evaluated the total consequence of these various factors. Third, dynamic monitoring of the SMA-RI was not performed in this study. Finally, The sample size was too small to evaluate the effects of different surgical types and different drugs on SMA-RI.

In conclusion, the increase of SMA-RI was associated with a higher lactate concentration and worse kinetics in patients after cardiac surgery. This may be related to the intestinal hypoperfusion. The SMA-RI may become one of the indicators that should be monitored to guide resuscitation. Future follow-up studies are needed to evaluate the prognosis of SMA-RI in these patients.

## Data Availability Statement

The original contributions presented in the study are included in the article/Supplementary Material, further inquiries can be directed to the corresponding author/s.

## Ethics Statement

The studies involving human participants were reviewed and approved by the Institutional Research and Ethics Committee of the Peking Union Medical College Hospital. The patients/participants provided their written informed consent to participate in this study.

## Author Contributions

YZ contributed to the conception and design of the study, acquisition of data, analysis, and interpretation of data, drafting of the article, and final approval of the version to be published. HH, NC, and XZ helped with the data collection and analysis. XW helped with building research ideas. DL guided the discussion. YL guided the research program. All authors contributed to the article and approved the submitted version.

## Funding

This work was supported by the Capital's Funds for Health Improvement and Research (2020-2-40111), the Medical and Health Science and Technology Innovation Project of the Chinese Academy of Medical Sciences (2019-12M-1-001), and the Excellence Program of Key Clinical Specialty of Beijing in 2020.

## Conflict of Interest

The authors declare that the research was conducted in the absence of any commercial or financial relationships that could be construed as a potential conflict of interest.

## Publisher's Note

All claims expressed in this article are solely those of the authors and do not necessarily represent those of their affiliated organizations, or those of the publisher, the editors and the reviewers. Any product that may be evaluated in this article, or claim that may be made by its manufacturer, is not guaranteed or endorsed by the publisher.
