# Supplementary material for: Resistance Index of the Superior Mesenteric Artery: Correlation With Lactate Concentration and Kinetics Prediction After Cardiac Surgery
Source: Front Med (Lausanne). 2021 Nov 24;8:762376. doi: 10.3389/fmed.2021.762376 (PMC8651699; doi:10.3389/fmed.2021.762376)
Supplement: Supplementary file 1 [file Data_Sheet_1.docx]

Supplementary Material

|  | | Admission | 2-hour | 6-hour | 12-hour |
| --- | --- | --- | --- | --- | --- |
| Lactate (mmol/L) | 4.10 (3.10 – 6.10) | | 4.40 (3.00 – 6.60) | 3.20 (2.10 – 4.90) | 1.90 (1.40 – 2.90) |
| Lactate kinetics (%) | / | | 4.40 (-18.90 – 12.70) | 3.20 (6.40 – 38.10) | 1.90 (36.60 – 66.70) |

**Supplementary Table 1.** Lactate concentrations and kinetics at various time points

The data are presented as median (interquartile range).

**Supplementary Table 2.** Characteristics of patients between high and low resistance index groups

| **Variables** | **High-RI**  **RI ≥ 0.83 (n = 37)** | **Low-RI**  **RI < 0.83 (n = 30)** |
| --- | --- | --- |
| Sex, n, female/male | 13/24 | 11/19 |
| Age (years) | 59.86±10.88 | 56.43±13.79 |
| Body Mass Index(kg/m^2^) | 22.82±1.91 | 23.11±2.00 |
| EF-preop (%) | 52.22±9.19 | 55.80±11.36 |
| MAP (mmHg) | 82.14±13.74 | 86.43±9.58 |
| HR (bpm) | 94.57±15.59 | 90.43±12.84 |
| CVP (mmHg) | 8.03±2.32 | 7.93±1.74 |
| HGB (g/L) | 102.5±34.61 | 95.73±31.26 |
| ScvO_2_ (%) | 70.01±9.26 | 71.47±6.58 |
| Pv-aCO_2_ (mmHg) | 5.28±2.04 | 4.50±1.64 |
| Catecholamine, n (%) | 34(91.9) | 30(100) |
| NE (µg/kg/min) | 0.21±0.27 | 0.16±0.16 |
| E (µg/kg/min) | 0.04±0.06 | 0.03±0.05 |
| VTI (cm/s) | 13.45±4.61 | 15.34±4.09 |
| Perfusion index | 1.28±1.33 | 1.45±1.07 |
| SOFA | 9.24±2.05 | 7.90±1.54 |
| APACHE-II | 16.19±3.67 | 15.90±4.59 |
| EURO-II | 4.53±3.93 | 2.69±1.73 |
| CPB (min) | 106.4±23.14 | 90.43±15.37 |
| Cross-clamp time (min) | 79.03±16.88 | 66.67±14.05 |
| CRRT, n (%) | 11(29.7) | 6(20.0) |
| **Procedures** |  |  |
| CABG surgery, n (%) | 10(27.0) | 12(40.0) |
| Valve surgery, n (%) | 21(56.8) | 17(56.7) |
| CABG + valve surgery, n (%) | 4(10.8) | 0(0) |
| Other, n (%) | 2(5) | 1(3.3) |
|  |  |  |

* *p* < 0.05. Data are presented as Frequency (percentage) or mean ± SD.

**Supplementary Figure 1**. Distribution of the resistance index of the superior mesenteric artery(SMA-RI)


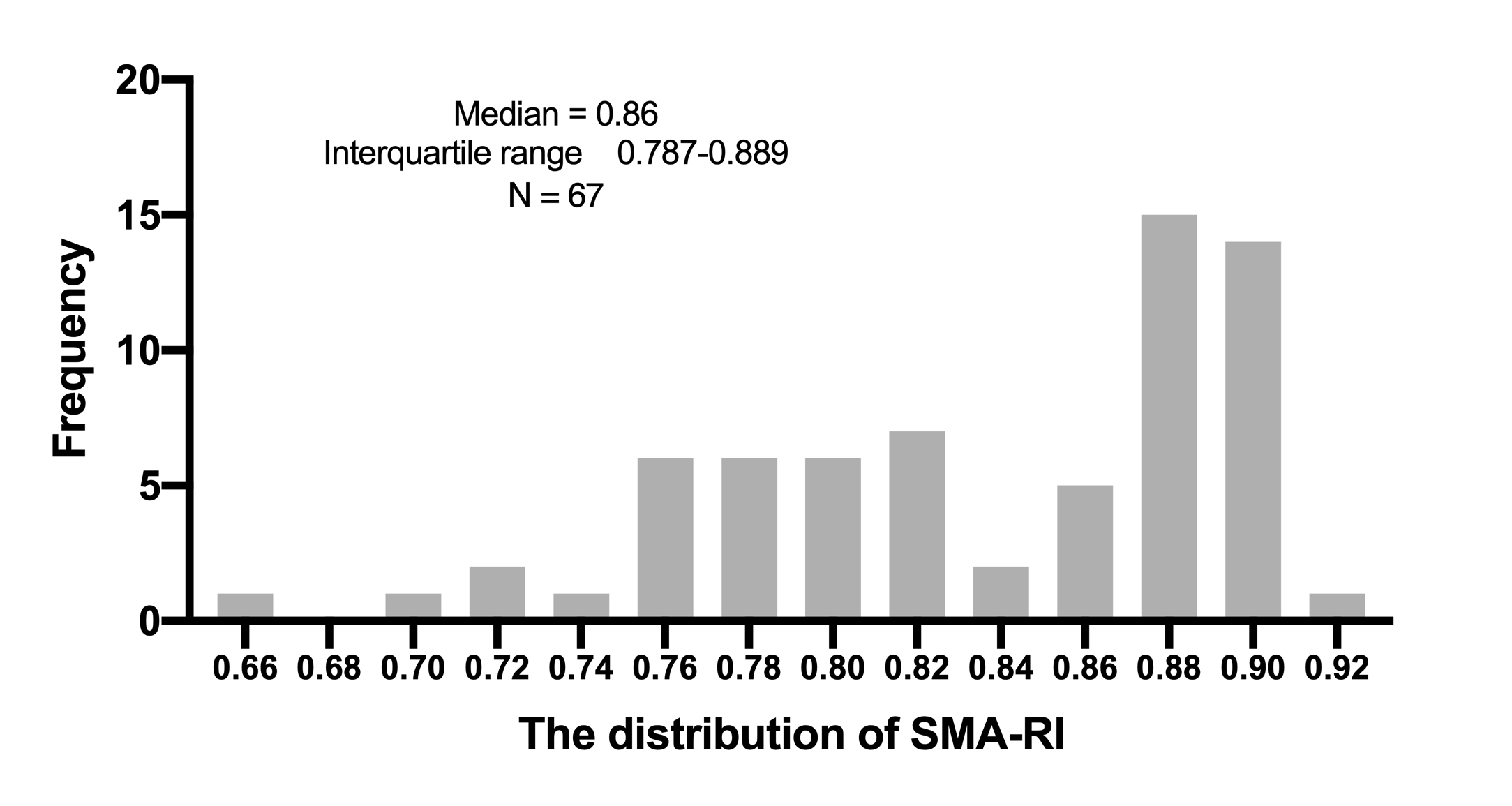


**Supplementary Figure 2**. Lactate concentration changes between high and low resistance index (RI) groups


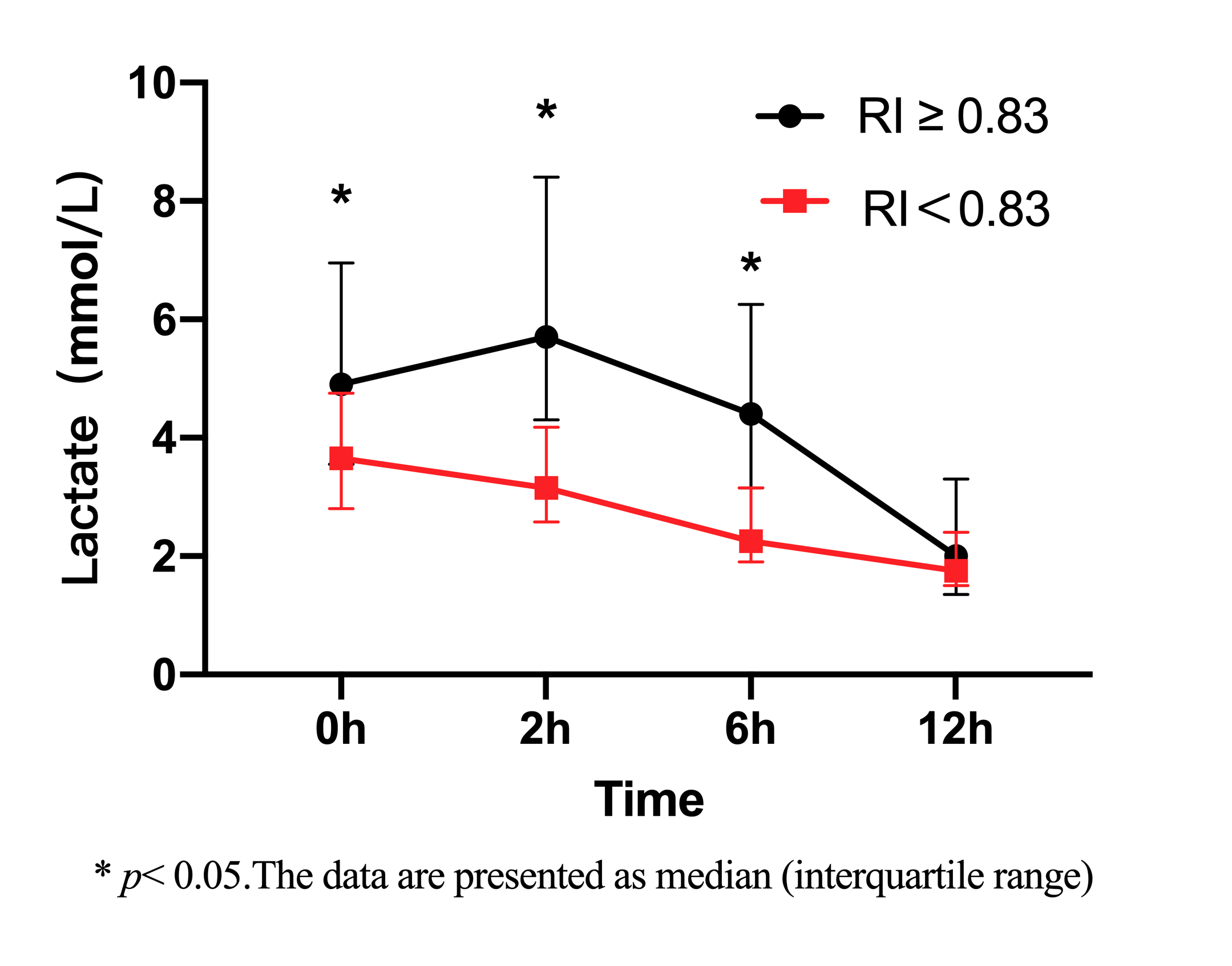


**Supplementary Figure 3**. Three-phase waveform of the superior mesenteric artery (SMA) blood flow


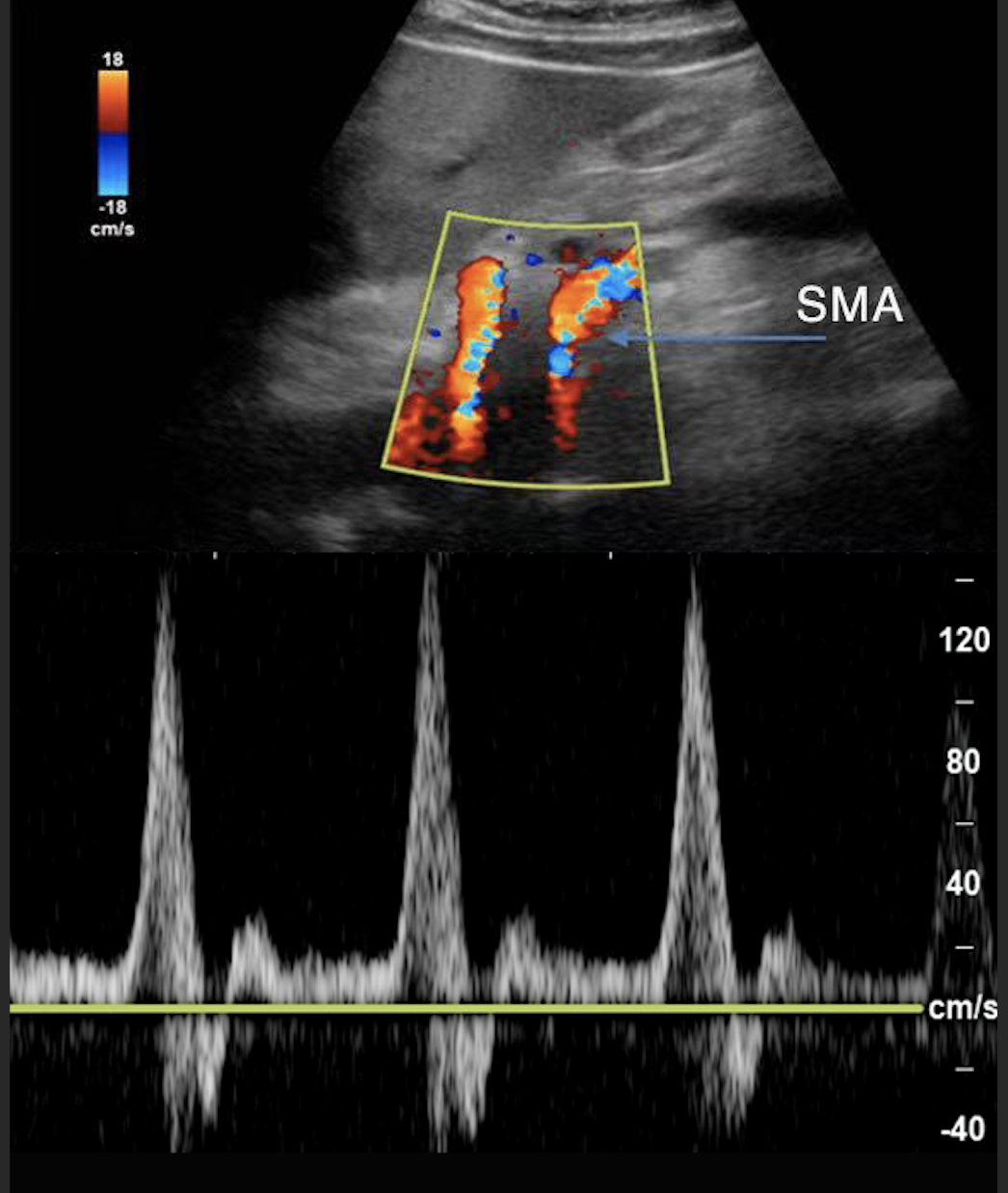


**
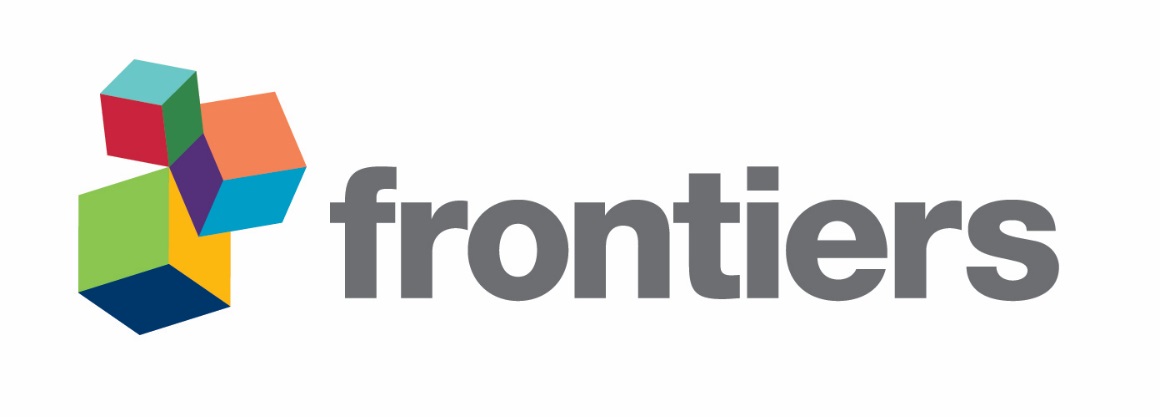
**
