# Supplementary figures and images for: Resistance Index of the Superior Mesenteric Artery: Correlation With Lactate Concentration and Kinetics Prediction After Cardiac Surgery
Source: Front Med (Lausanne). 2021 Nov 24;8:762376. doi: 10.3389/fmed.2021.762376 (PMC8651699; doi:10.3389/fmed.2021.762376)

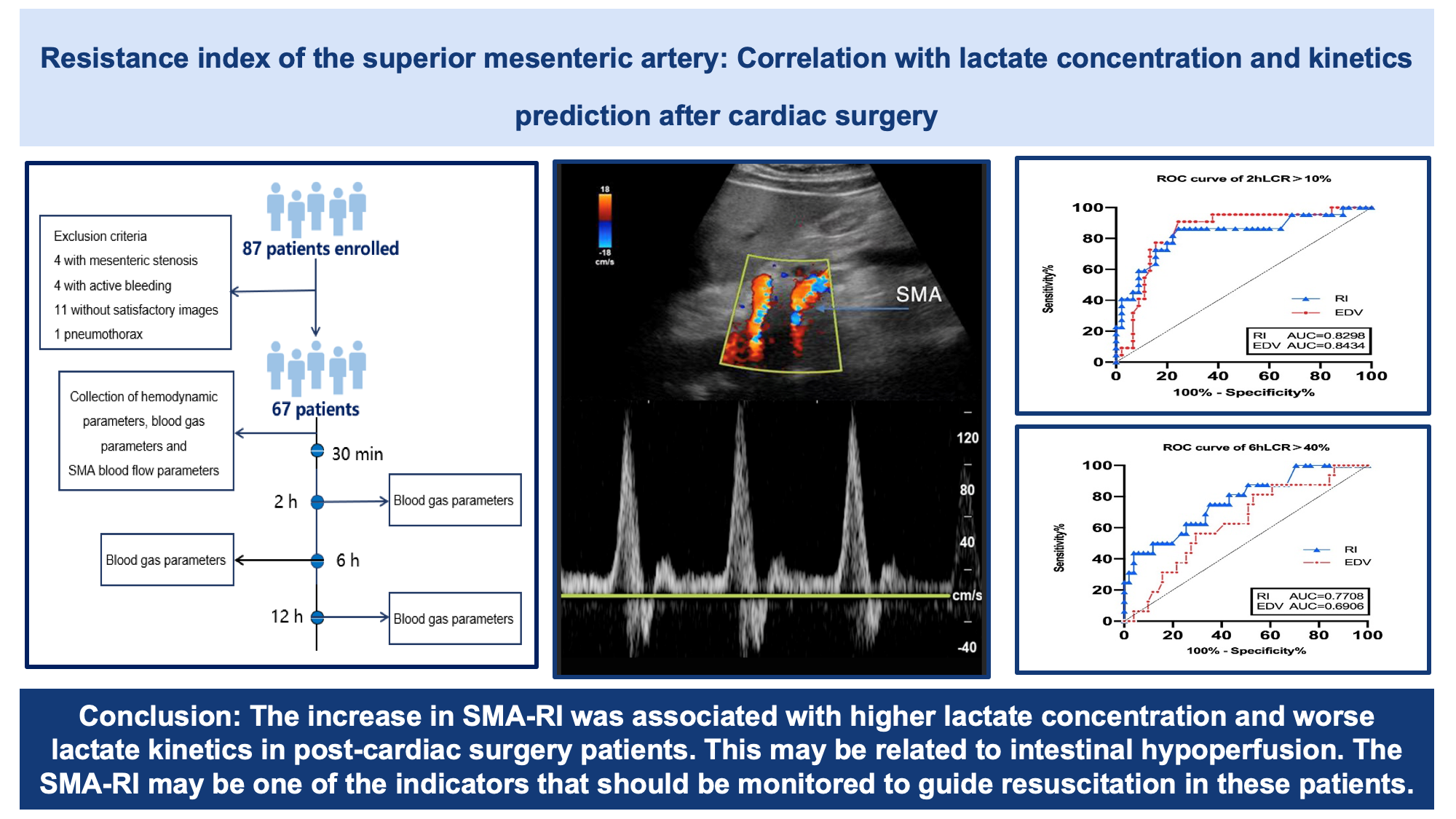

Supplement: Supplementary file 2 [file Image_1.TIFF]
